# Prevalence of testosterone deficiency in HIV-infected men under antiretroviral therapy

**DOI:** 10.1186/s12879-016-1892-5

**Published:** 2016-11-03

**Authors:** Ana Rita Gomes, Pedro Souteiro, Carolina Germana Silva, Bernardo Sousa-Pinto, Francisco Almeida, António Sarmento, Davide Carvalho, Paula Freitas

**Affiliations:** 1 Faculty of Medicine, University of Porto, Alameda Prof. Hernâni Monteiro, 4200-319 Porto, Portugal; 2 Endocrinology, Diabetes and Metabolism Department, Centro Hospitalar São João, Porto, Portugal; 3 Department of Health Information and Decision Sciences, Faculty of Medicine (CIDES), University of Porto, Rua Dr. Placido da Costa, 4200-450 Porto, Portugal; 4 Center for Health Technology and Services Research (CINTESIS), Faculty of Medicine, University of Porto, Rua Dr. Placido da Costa, 4200-450 Porto, Portugal; 5 Infectious Diseases Department, Centro Hospitalar São João, Faculty of Medicine, University of Porto, Porto, Portugal; 6 Faculty of Medicine, i3S - Instituto de Investigação e Inovação em Saúde, University of Porto, Porto, Portugal

**Keywords:** HIV infection, Hypogonadism, Lipodystrophy, Cardiovascular risk

## Abstract

**Background:**

The prevalence of hypogonadism in HIV-infected patients is still a matter of debate as there is no standardized consensual diagnostic method. In addition, the etiology and endocrine/metabolic implications of hypogonadism in this population remain controversial. This study aims to determine the prevalence of testosterone deficiency in a single-site hospital and to evaluate its association with potential risk factors, lipodystrophy, metabolic syndrome, and cardiovascular risk.

**Methods:**

This study analyzed 245 HIV-infected men on combined antiretroviral therapy. Patients with low total testosterone (TT) levels (<2.8 ng/mL) and/or low calculated free testosterone (FT) levels (<6.5 ng/dL) were considered testosterone deficient. According to their LH and FSH levels, patients were classified as having hypogonadotropic or hypergonadotropic dysfunction. Other clinical, anthropometric, and analytic parameters were also collected and analyzed.

**Results:**

The prevalence of testosterone deficiency in our population was 29.4 %. Among them, 56.9 % had hypogonadotropic dysfunction and 43.1 % presented with hypergonadotropic dysfunction. Patients with testosterone deficiency were older (*p* < 0.001), had higher HbA1c levels (*p* = 0.016) and higher systolic blood pressure (*p* = 0.007). Patients with lower testosterone levels had higher prevalence of isolated central fat accumulation (*p* = 0.015) and had higher median cardiovascular risk at 10 years as measured by the Framingham Risk Score (*p* = 0.004) and 10-Year ASCVD risk (*p* = 0.002).

**Conclusions:**

The prevalence of testosterone deficiency in this HIV population is high, with hypogonadotropic dysfunction being responsible for the majority of cases. Testosterone deficiency might predispose to, or be involved, in the pathogenesis of HIV-associated lipodystrophy. Patients with low testosterone levels have higher cardiovascular risk, highlighting the importance of early diagnosis of this condition.

## Background

Several endocrine abnormalities, like hypogonadism, thyroid dysfunction, adrenal insufficiency, and diabetes were described as more prevalent in HIV patients in the pre-combined antiretroviral therapy (cART) era. They were mainly associated to HIV-related opportunistic infections and advanced multi-organ failure [1–3]. With the advent of highly active antiretroviral therapy in the 1990s, some of these metabolic diseases have waned but hypogonadism and diabetes remain frequent endocrinopathies in these patients [4].

Gonadal dysfunction leads to sexual impairment, bone and muscle mass loss, changes in body fat mass, fatigue, cognitive impairment, depressed mood and anemia, affecting the overall quality of life [1, 5, 6]. After assessing testosterone levels, additional tests are needed to determine whether hypogonadism is primary (hypergonadotropic), or secondary (hypogonadotropic) [1, 7]. According to previous studies in this population, hypogonadotropic dysfunction is responsible for the majority of cases, while primary disease is seen less frequently (occasionally, patients may have a combination of both). [8]. Overall, the causes of testosterone deficiency among HIV-infected men remain controversial, and there is lack of studies concerning the etiology and metabolic and endocrine consequences of this condition [1]. Additionally, the prevalence of testosterone deficiency in the HIV population is not well defined, ranging from 16 to 54 %, as slightly different criteria are adopted in different studies [4, 6, 9].

Therefore, this study aims to determine the prevalence of testosterone deficiency in a population of HIV-infected men under cART, as well as the prevalence of hypo- and hypergonadotropic dysfunction. Additionally, it aims to assess the association between testosterone deficiency and potential risk factors, and to evaluate the relationship between lower testosterone levels, lipodystrophy, metabolic syndrome and cardiovascular risk.

## Methods

### Subjects

Two-hundred and forty five adult men with serologically documented HIV infection on cART were evaluated at the Endocrinology Outpatient Department of Centro Hospitalar São João (a tertiary center in Northern Portugal). Patients were consecutively referred from the Infectious Diseases Clinic between January/2008 to December/2014, and were enrolled in the first visit.

Patients with acute or severe illness, previous treatment with androgen-derived agents and concurrent endocrinopathy (e.g. uncontrolled hypothyroidism, adrenal insufficiency, hyperprolactinemia, known pituitary disease) were excluded.

### Clinical assessment

For all patients, the following information was collected, using a standardized protocol: age, time since HIV diagnosis, risk factor for HIV transmission, type and duration of cART exposure, CD4^+^ count, plasmatic HIV RNA load, smoking history (current, past, or never) and alcohol consumption (yes or no). HIV stages were defined using the Centers for Disease Control and Prevention’s (CDC) classification into asymptomatic (A), symptomatic (B) and AIDS (C) [10].

Height was measured in the standing position, using a wall stadiometer (Holtain Limited Crymych, Dyfed®), and body weight was measured using the TANITA (Tanita®, model TBF 300) scale. Circumferences of neck, waist, hip, thigh and arm were measured, as previously described, and blood pressure (BP) was measured using the current recommendations [11, 12].

Clinical lipodystrophy was assessed by both patient and practitioner, and was defined as peripheral lipoatrophy, with or without central fat accumulation [12]. Abdominal prominence (central obesity) was defined by a waist circumference >102 cm, as defined by NCEP-ATP III modified criteria for metabolic syndrome [13]. All clinical assessments were performed by the same observer. Patients were classified into four different groups: (1) No lipodystrophy (patients without clinical lipoatrophy and without abdominal prominence); (2) Isolated central fat accumulation (patients without clinical lipoatrophy and with abdominal prominence); (3) Isolated lipoatrophy (patients with clinical lipoatrophy and without abdominal prominence), and; (4) Mixed forms of lipodystrophy (patients with clinical lipoatrophy and with abdominal prominence) [11].

### Laboratory analysis

An early morning venous blood sample was taken after a 12-hour overnight fast. All samples were analyzed at the central laboratory of our hospital. Total testosterone (TT), sex hormone binding globulin (SHBG), luteinizing hormone (LH), and follicle-stimulating hormone (FSH) were determined using Cobas e411 (*Roche Diagnostic*). Glycosylated hemoglobin (HbA1c) was measured by Variant II Turbo (*Bio-Rad*). To evaluate lipid profile, we measured total cholesterol, LDL cholesterol (LDL-C), HDL cholesterol (HDL-C) and triglycerides levels, using Olympus AU5400 (*BecKman Coulter*). The CD4+ cell count (×10^6^ cell/L) and plasma HIV RNA loads were assessed by flow cytometry and by a quantitative reverse transcriptase polymerase chain reaction (*Roche Diagnostic*), respectively.

### Criteria for the definition of gonadal status

Testosterone deficiency was defined based on a serum total testosterone (TT) below 2.8 ng/mL and/or a calculated free testosterone levels (FT), according to Vermeulen formula, below 6.5 pg/mL [14].

According to the normal ranges for serum LH and FSH, as defined by our central laboratory (1.7–8.3 mUI/mL and 1.5–12.4 mUI/mL, respectively), patients were assigned as having hypogonadotropic (low or normal LH and/ or FSH) or hypergonadotropic dysfunction (high LH and/ or FSH). Patients were classified as having high sex hormone-binding globulin (SHBG) according to the threshold value of 48.4 nmol/L.

### Metabolic syndrome definition and cardiovascular risk scores

We defined Metabolic Syndrome (MS) according to the modified NCEP-ATP III criteria, requiring the presence of three of the following risk factors: (1) elevated waist circumference (>102 cm); (2) elevated triglycerides (≥150 ml/dL or specific treatment); (3) reduced HDL-C levels (<40 mg/dL), (4) elevated blood pressure (>130/85 mmHg or treatment with anti-hypertensive drugs), and; (5) elevated fasting glucose (≥100 mg/dL or use of glucose-lowering drugs) [13].

The cardiovascular disease (CVD) risk at 10 years was measured by the Framingham Risk Score (FRS), using a sex-specific algorithm that incorporates age, treated, and untreated systolic blood pressure, HDL-C, total cholesterol, glucose, and smoking status [15]. Based on the Adult Treatment Panel III (ATP III) guidelines, individuals were stratified into four risk categories: (1) Low risk (0–1 CVD risk factors); (2) moderate risk (≥2 CVD risk factors and a 10-year CVD risk <10 %); (3) moderately high risk (≥2 CVD risk factors and a 10-year CVD risk 10–20 %), and; (4) high risk (CHD or CHD equivalent or ≥2 CVD risk factors and a 10-year CVD risk >20 %) [16].

The 10-year and lifetime atherosclerotic cardiovascular disease (ASCVD) risk scores were calculated based on the American College of Cardiology (ACC)/ American Heart Association’s (AHA) guidelines. For all patients, we used a sex, ethnic group and age-specific multivariable risk factor algorithm that incorporates total cholesterol, HDL-C, untreated systolic blood pressure, diabetes history, and smoking status [17].

### Statistical analysis

Categorical variables were presented as absolute frequencies and percentages, and were analyzed using Chi-square or Fisher’s exact test. Continuous variables were presented as medians and interquartile ranges, and were analyzed using the Mann–Whitney *U* test. *P* values <0.05 were considered to be statistically significant. All statistical analysis was performed using SPSS version 22.0 (SPSS, Inc., Chicago, IL).

## Results

A total of 245 HIV-infected males on cART were evaluated, and 29.4 % were found to have testosterone deficiency, with a median of TT and calculated FT of 2.6 ng/mL and 5.2 ng/dL, respectively. Among the patients with reduced TT, 50 % had high SHBG levels.

In the group of patients with testosterone deficiency, 56.9 % had hypogonadotropic and 43.1 % had hypergonadotropic dysfunction. When different clinical, anthropometric and analytic variables were analyzed regarding these two categories of testosterone deficiency, patients with elevated gonadotrophins presented a higher frequency of NRTI use (100 % *vs*. 86.7 %; *p* = 0.028) and lower CD4+ counts (336.0 *vs*. 449.0; *p* = 0.030). No other differences were found between the two groups of patients.

The baseline characteristics of our study population are shown in Table 1. Patients with testosterone deficiency were older than the remainder (median age of 51.5 *versus* 48.0 years; *p* < 0.001). No significant differences were observed regarding the risk factors for HIV transmission, stages of CDC criteria, length of HIV infection, and cART, CD4+, viral count, viral suppression, and class of antiretroviral drugs used.

**Table 1:** Participants baseline characteristics, according to the presence of testosterone deficiency

	Total (*n* = 245)	Testosterone deficiency (*n* = 72)	Without testosterone deficiency (*n =* 173)	*p* value
Age [years, median (IQR)]	48.0 (15.0)	51.5 (11.8)	48.0 (7.3)	<0.001^a^
Duration of HIV infection [years, median (IQR)]	8.0 (6.0)	6.0 (8.0)	8.0 (6.0)	0.567^a^
HIV stage [n (%)]^d^				
A	122 (52.1)	31 (44.9)	91 (55.2)	0.157^b^
B	12 (5.1)	6 (8.7)	6 (3.6)	
C	100 (42.7)	32 (46.4)	68 (41.2)	
cART [years, median (IQR)]	6.0 (7.0)	4.5 (8.8)	7.0 (6.0)	0.447^a^
NRTI [n (%)]	213 (86.9)	67 (31.5)	146 (84.4)	0.067^c^
NNRTI [n (%)]	104 (42.4)	33 (45.8)	71 (41.0)	0.489^b^
PI [n (%)]	119 (48.6)	33 (45.8)	86 (49.7)	0.580^b^
FI [n (%)]	1 (0.4)	0	1 (0.6)	0.999^c^
II [n (%)]	16 (6.5)	6 (8.3)	10 (5.8)	0.570^c^
Viral load (<50) [n (%)]	131 (82.4)	33 (76.7)	98 (84.5)	0.255^b^
CD4 count [cells/mm^3^, median (IQR)]	477.0 (327.3)	501.0 (351.5)	526.5 (286.8)	0.157^a^
HIV risk factor [n (%)]				
Injecting drug user	55 (23.7)	18 (26.5)	37 (22.6)	0.561^b^
Heterosexual contact	118 (50.9)	37 (54.4)	81 (49.4)	
Homosexual contact	50 (21.6)	11 (16.2)	39 (23.8)	
Others	9 (3.9)	2 (2.9)	7 (4.3)	

*cART* combined antiretroviral therapy, *NRTI* nucleoside reverse transcriptase inhibitor, *NNRTI* non-nucleoside reverse transcriptase inhibitor, *PI* protease inhibitor, *FI* fusion inhibitor, *II* integrase inhibitor

^a^Mann-Whitney *U* test; ^b^Chi-square test, ^c^Fisher test; ^d^Information only available for 234 patients

Table 2 shows the clinical characteristics of the population studied. Patients with testosterone deficiency had a significantly higher median waist circumference (99.5 cm *versus* 93.0 cm; *p* = 0.030). Lower testosterone individuals also presented a higher median HbA1c percentage (5.5 % *versus* 5.3 %; *p* = 0.016) and a higher median systolic blood pressure (133.5 mmHg *versus* 130.0 mmHg; *p* = 0.007). There were no significant differences between the two groups concerning weight, lipid profile, neck, upper-arm, abdominal, and hip circumferences, smoking status, alcohol consumption and frequency of metabolic syndrome. When patients were stratified into four groups of fat distribution (presence or not of clinical lipoatrophy and abdominal prominence), patients with lower testosterone levels had a higher percentage of isolated central fat accumulation (18.1 % *vs*. 7.5 %; *p* = 0.015).

**Table 2:** Participants clinical characteristics according to the presence of testosterone deficiency

	Testosterone deficiency (*n* = 72)	Without testosterone deficiency (*n* = 173)	*p* value
Weight [kg, median (IQR)]	76.3 (25.9)	71.3 (16.2)	0.857^a^
Height [cm, median (IQR)]	171.0 (8.0)	169.0 (7.5)	0.204^a^
BMI [(kg/m^2^), median (IQR)]	26.5 (16.8)	25.2 (5.4)	0.160^a^
Underweight [n (%)]	5 (7.0)	6 (3.6)	0.028^b^/0.155^c^
Normal weight [n (%)]	26 (36.3)	78 (46.2)	
Overweight [n (%)]	23 (32.4)	67 (39.6)	
Obesity [n (%)]	17 (23.9)	18 (10.7)	
Neck circumference [cm, median (IQR)]	40.0 (4.8)	39.0 (3.0)	0.196^a^
Upper-arm circumference [cm, median (IQR)]	28.3 (3.8)	28.0 (3.8)	0.197^a^
Abdominal circumference [cm, median (IQR)]	98.0 (13.8)	93.0 (8.6)	0.492^a^
Waist circumference [cm, median (IQR)]	99.5 (19.8)	93.0 (13.5)	0.030^a^
Hip circumference [cm, median (IQR)]	47.0 (6.0)	47.5 (6.0)	0.906^a^
Metabolic syndrome [n (%)]	33 (45.8)	69 (39.9)	0.390^b^
Blood pressure [n (%)]	30 (41.7)	69 (40.1 %)	0.822^b^
Systolic blood pressure [median (IQR)]	133.5 (40.0)	130.0 (30.0)	0.007^a^
Diastolic blood pressure [median (IQR)]	79.0 (25.0)	80.0 (15.0)	0.756^a^
Smoking history [n (%)]			
Current	21 (30.4)	72 (42.6)	0.127^b^
Former	25 (36.2)	42 (24.9)	
Never	23 (33.3)	55 (32.5)	
Alcohol consumption [n (%)]	41 (59.4)	77 (45.6)	0.052^b^
Total cholesterol [mg/dL, median (IQR)]	210.0 (69.0)	221.0 (70.5)	0.668^a^
LDL cholesterol [mg/dL, median (IQR)]	129.0 (37.0)	129.0 (72.8)	0.573^a^
HDL cholesterol [mg/dL, median (IQR)]	43.5 (13.3)	42.5 (13.3)	0.125^a^
Triglycerides [mg/dL, median (IQR)]	215.0 (215.3)	218.0 (162.0)	0.631^a^
HbA1c [%, median (IQR)]	5.5 (1.7)	5.3 (1.0)	0.016^a^
Total testosterone [ng/mL, median (IQR)]	2.6 (2.6)	5.9 (2.7)	<0.001^a^
Free testosterone [ng/dL, median (IQR)]	5.2 (1.5)	9.1 (3.6)	<0.001^a^
SHBG [nmol/L, median (IQR)]	35.2 (44.6)	46.6 (29.6)	0.083^a^
LH [mIU/mL, median (IQR)]	5.0 (4.9)	5.2 (3.2)	0.001^a^
FSH [mIU/mL, median (IQR)]	6.7 (7.1)	5.1 (4.1)	<0.001^a^
FRS [median (IQR)]	13.0 (5.0)	12.0 (6.0)	0.004^a^
<10 [n (%)]	16 (22.2)	66 (38.2)	0.008^b^/0.003^c^
10–20 [n (%)]	48 (66.7)	101 (58.4)	
>20 [n (%)]	8 (11.1)	6 (3.5)	
10-Year ASCVD risk [%, median (IQR)]	7.1 (6.5)	6.8 (8.3)	0.002^a^
Lifetime ASCVD risk [n (%)]			
<40 %	4 (10.0)	11 (8.0)	0.789^b^
40–50 %	22 (55.0)	84 (60.9)	
>50 %	14 (35.0)	43 (31.2)	
Lipodistrophy [n (%)]	39 (56.5)	96 (58.5)	0.776^b^
Waist circumference (>102 cm) [n(%)]	23 (31.9)	30 (17.3)	0.011^b^
No lipodystrophy [n (%)]	20 (27.8)	59 (34.3)	0.321^b^
Isolated central fat accumulation [n (%)]	13 (18.1)	13 (7.5)	0.015^b^
Isolated lipoatrophy [n (%)]	29 (40.3)	83 (48.3)	0.254^b^
Mixed forms of lipodystrophy [n (%)]	10 (13.9)	17 (9.8)	0.355^b^

*BMI* body mass index, *LDL* low density lipoprotein, *HDL* high density lipoprotein, *HbA1c* glycosylated hemoglobin, *SHBG* sex hormone binding globulin, *LH* luteinizing hormone, *FSH* follicle stimulating hormone, *FRS* Framingham risk score, *ASCVD* atherosclerotic cardiovascular disease

^a^Mann-Whitney *U* test; ^b^Chi-square test; ^c^Linear by linear association test

Patients with testosterone deficiency had a significantly higher risk of CVD at 10 years, as measured by the FRS (median score of 13.0 and 12.0; *p* = 0.004). Those patients also presented a significantly higher 10-Year risk of CVD with the ASCVD risk score (7.1 *versus* 6.8; *p* = 0.002), although no significant differences were found concerning lifetime ASCVD risk (*p* = 0.789).

## Discussion

The introduction of cART has led to a decreased prevalence of hypogonadism among HIV-infected men. This change is probably related to the reduced frequency of end-stage AIDS, opportunistic infections, chronic immune activation and HIV-associated wasting syndrome. However, hypogonadism remains an important problem in HIV-infected patients, where higher rates of this condition are found compared to the general population, even in patients with early stages of the disease [1, 18]. As previously mentioned, the prevalence of hypogonadism in this population is still a matter of debate, ranging from 16 to 54 % in different reports [4, 6, 9]. This discrepancy might be due to a lack of standardized, validated methods for its assessment, and to a lack of universal agreement in a clinical and/or biochemical definition for hypogonadism [19]. In our study, we obtained a prevalence of testosterone deficiency of 29.4 % in a population of 245 HIV-infected men on cART. It has been recognized that testosterone levels may be difficult to interpret in HIV-infected patients, as they tend to have a higher concentration of SHBG, which may increase TT values [1, 18, 20]. Therefore, following the recommendations to use FT levels in individuals with HIV infection, we used TT and FT calculated by the Vermeulen formula to define testosterone deficiency, in order to avoid false negatives that would possible occur if only TT levels had been used. Accordingly, if only serum TT had been used, some patients with low testosterone levels would have been missed as 50 % of them had high SHBG levels [14, 21].

Regarding the categories of testosterone deficiency, our results shows that hypogonadotropic dysfunction was responsible for the majority of cases. This is in accordance with several studies that report a progressive change in the etiology of testosterone deficiency in HIV-infected patients. Publications in the pre-cART era reported higher prevalences of gonadal dysfunction caused by direct involvement by the virus with a reduction in the Leydig cell number [22]. Recent investigations, after the appearance of more effective therapy, showed a relatively higher preponderance of secondary dysfunction [6]. Several different mechanisms have been suggested to explain the effect on the hypothalamic-pituitary-gonadal (HPG) axis, including poor health status, undernutrition, frailty, anti-retroviral drugs, the virus *per se*, opportunistic infections and increased visceral adiposity [8].

Consistently with other studies, the presented data shows that the prevalence of testosterone deficiency increases with age, as it happens in the general population However, there was no significant impact of the HIV transmission risk factor and sexual orientation on testosterone levels, confirming the findings of other groups [1]. With regards to the antiretroviral agents, our study did not find any difference between patients with low testosterone levels and the remaining ones among the five studied pharmacologic groups. In fact, previous studies show conflicting results in this area [1, 7, 18].

We have shown that 18.1 % of patients with testosterone deficiency presented visceral obesity (as measured by waist circumference >102 cm), contrasting to 7.5 % of eugonadal individuals. The lipodystrophy syndrome, which has a reported prevalence of 41 % in HIV-infected patients, is a well-known adverse effect of cART. It comprises fat redistribution abnormalities, insulin resistance and dyslipidemia [7, 23]. However, the impact of cART on the HPG axis is still not completely understood, and little is known about the influence of sex hormone levels on the development of lipodystrophy [23]. Testosterone is one of the major determinants of regional distribution of fat and body composition, favoring the accumulation of visceral fat. The underlying pathophysiological mechanisms include an increase in tissue sensitivity to glucocorticoids, reduction of adipokines, and a reduction of peroxisome proliferator-activated receptor (PPAR-γ) activity [24]. Testosterone role in insulin sensitivity in HIV-lipodystrophy has also been proposed since several studies have shown that low levels of serum testosterone correlate not only with central fat accumulation but also with higher insulin levels [7].

HIV-infected patients also present an increased cardiovascular risk when compared with the general population. The pathogenesis of CVD in these patients is complex, and several factors appear to be involved, including androgen dysfunction [7, 25]. Low testosterone levels are independently associated with cardiovascular risk and are independent predictors of mortality [19, 26]. Accordingly, in our study, HIV patients with lower testosterone levels had a significantly higher 10-year risk of coronary heart disease, as measured by the FRS and 10-Year ASCVD. We could hypothesize that men with testosterone deficiency, besides being older, are at additional CV risk due to the higher frequency of hyperglycemia, abdominal obesity and hypertension as described in our results. Regan et al. compared the proportion of HIV patients with high predicted 10-year CVD risk using the FRS *versus* the ASCVD risk prediction algorithm [27]. The authors reported that the ASCVD algorithm classified a larger proportion of HIV patients as high-risk, compared to the FRS. This is consistent with our results, since a higher percentage of individuals were classified as having a high-risk by the ASCVD algorithm, independently of their testosterone status. Nevertheless, no significant differences were found in the lifetime risk of cardiovascular disease. This discrepancy can possibly be explained by the fact that only patients aged between 20 and 59 years-old could be included in this score. Regardless, we should mention that both the FRS and ASCVD appear to underestimate the actual risk of events, as reported by Thompson-Paul et al. [28]. Therefore, risk assessment remains reliant on the use of traditional risk factors, despite their inherent limitations. To better estimate CVD risk, different models including additional HIV-specific risk factors, such as immunologic or virologic status, may need to be considered [25, 28]. Incorporation of imaging studies assessing subclinical atherosclerosis in risk algorithms for HIV patients may add useful information in the future [25].

This study has some limitations that are inherent to the observational design and the cross-sectional nature of our analyses. In addition, patients with testosterone deficiency diagnosed prior to HIV infection were not excluded. Consequently, conclusions regarding causality cannot be made with certainty. Although we have included all patients referred to our department, bias in the referral cannot be excluded, since some patients could have been referred because they already had testosterone deficiency or metabolic disorders related to cART. Therefore, we might have selected a study population where metabolic and endocrine complications were over-represented.

Our study has some strong points, including the fact that the study was performed in a highly experienced unit in the assessment of metabolic and body fat abnormalities in HIV-infected patients, with all clinical assessments being performed by the same investigator. Finally, another positive point was the fact that our sample size was relatively large when compared to other studies performed in this area.

## Conclusions

The prevalence of testosterone deficiency in HIV-infected men is high, with hypogonadotropic dysfunction being responsible for the majority of cases. Patients with lower testosterone levels were older, had poorer glycemic control, higher systolic blood pressure and increased waist circumference. Our results corroborate the hypothesis that testosterone deficiency may predispose or might be involved in the pathogenesis of HIV-associated lipodystrophy. This has a significant clinical impact, since both conditions are common in this population and are associated with a negative impact on the quality of life and with poor adhesion to therapy. Additionally, this study suggests that patients with lower testosterone levels have higher cardiovascular risk scores, highlighting the importance of early diagnosis of this condition.

## Abbreviations

ACC: American college of cardiology
AHA: American heart association
AIDS: Acquired Immune Deficiency Syndrome
ASCVD: Atherosclerotic cardiovascular disease
BMI: Body mass index
cART: Combined antiretroviral therapy
CDC: Centers for Disease Control and Prevention
FI: Fusion inhibitor
FRS: Framingham risk score
FSH: Follicle stimulating hormone
FT: Free testosterone
HbA1c: Glycosylated hemoglobin
HDL: High density lipoprotein
HIV: Human immunodeficiency virus
HPG: Hypothalamic-pituitary-gonadal
IDF: International diabetes federation
II: Integrase inhibitor
LDL: Low density lipoprotein
LH: Luteinizing hormone
MS: Metabolic syndrome
NNRTI: Non-nucleoside reverse transcriptase inhibitor
NRTI: Nucleoside reverse transcriptase inhibitor
PI: Protease inhibitor
SHBG: Sex hormone binding globulin
TT: Total testosterone
